# Plasticity of the Intrinsic Period of the Human Circadian Timing System

**DOI:** 10.1371/journal.pone.0000721

**Published:** 2007-08-08

**Authors:** Frank A.J.L. Scheer, Kenneth P. Wright, Richard E. Kronauer, Charles A. Czeisler

**Affiliations:** Division of Sleep Medicine, Department of Medicine, Brigham & Women's Hospital and Division of Sleep Medicine, Harvard Medical School, Boston, Massachusetts, United States of America; Duke University, United States of America

## Abstract

Human expeditions to Mars will require adaptation to the 24.65-h Martian solar day-night cycle (sol), which is outside the range of entrainment of the human circadian pacemaker under lighting intensities to which astronauts are typically exposed. Failure to entrain the circadian time-keeping system to the desired rest-activity cycle disturbs sleep and impairs cognitive function. Furthermore, differences between the intrinsic circadian period and Earth's 24-h light-dark cycle underlie human circadian rhythm sleep disorders, such as advanced sleep phase disorder and non-24-hour sleep-wake disorders. Therefore, first, we tested whether exposure to a model-based lighting regimen would entrain the human circadian pacemaker at a normal phase angle to the 24.65-h Martian sol and to the 23.5-h day length often required of astronauts during short duration space exploration. Second, we tested here whether such prior entrainment to non-24-h light-dark cycles would lead to subsequent modification of the intrinsic period of the human circadian timing system. Here we show that exposure to moderately bright light (∼450 lux; ∼1.2 W/m^2^) for the second or first half of the scheduled wake episode is effective for entraining individuals to the 24.65-h Martian sol and a 23.5-h day length, respectively. Estimations of the circadian periods of plasma melatonin, plasma cortisol, and core body temperature rhythms collected under forced desynchrony protocols revealed that the intrinsic circadian period of the human circadian pacemaker was significantly longer following entrainment to the Martian sol as compared to following entrainment to the 23.5-h day. The latter finding of after-effects of entrainment reveals for the first time plasticity of the period of the human circadian timing system. Both findings have important implications for the treatment of circadian rhythm sleep disorders and human space exploration.

## Introduction

The daily pattern of sleep and wakefulness is regulated by homeostatic and circadian processes [1], [2]. The master circadian pacemaker, located in the suprachiasmatic nucleus of the anterior hypothalamus (SCN), orchestrates near-24-h rhythms in physiology and behavior in mammals, including the sleep-wake cycle [3], [4]. Environmental light exposure can shift the phase of the circadian timing system depending on properties of the light exposure such as timing, intensity, duration, and wavelength [5]–[8]. Entrainment at a normal phase angle requires daily phase shifts that compensate for the difference between the intrinsic circadian period and the imposed light-dark cycle (T-cycle) and result in the elevated production of the soporific hormone melatonin during the scheduled sleep episode and minimal melatonin production during the scheduled wake episode. Failure of proper entrainment of the circadian time keeping system to the desired sleep-wake cycle disturbs sleep and impairs cognitive function [9], [10]. In the present study, we tested two hypotheses: (1) that the human circadian pacemaker can be entrained at a normal phase angle to non-24-h rest-activity cycles; and (2) that the period of the human circadian pacemaker would show plasticity following entrainment to non-24-h rest-activity cycles.

It was not known whether the human circadian timing system could be entrained at a normal phase angle to both a shorter-than and longer-than 24-h rest-activity cycle without relying on exposure to artificial bright light, which is not available on any spacecraft built to date. Given initial reports that the period of the human circadian pacemaker was close to 25 h [11], [12], it had been presumed that the human circadian sleep-wake cycle would readily synchronize to the 24.65-h solar day-night cycle (sol) on the planet Mars. However, the more recent discovery that the intrinsic period of the human circadian pacemaker is very close to 24 h (i.e., 24.18 h) [13] led to the subsequent demonstration that the human circadian pacemaker is unable to entrain to either the Martian day (24.65 h) or the 23.5-h sleep-wake cycle often required of astronauts during space shuttle missions under the rather dim lighting conditions to which astronauts are commonly exposed [10], [14]. Many other factors, in addition to dim light aboard space crafts, may contribute to circadian misalignment during space flight. Windows and exposure to sunlight of future mission astronauts may be restricted during long duration space flights because of dangerous particle radiation while traveling outside the Earth's protective magnetic field and while on Mars because of radiation, sun-blocking dust storms, and extreme temperatures. Thus, astronauts may need to rely largely on artificial light that is costly to generate, which is the primary reason why ambient light is so dim on the International Space Station and aboard the U.S. Space Shuttle fleet. Furthermore, to achieve maximal phase resetting, a very bright light exposure (∼10,000 lux; 27 W/m^2^) should be timed close to the circadian core body temperature minimum, which would interfere with normal timing of the sleep episode. In the present study, we therefore evaluated whether exposure to only moderately bright light limited to part of the scheduled wake episode could entrain the human circadian pacemaker at a normal phase angle to the Martian sol and to a 23.5-hour cycle. Given that the resetting effects of light are both phase-dependent [7], [15] and intensity-dependent [5], [6], we used Kronauer's mathematical model of the resetting effect of light on the human circadian pacemaker [16] to design a temporal pattern of environmental light exposure consisting of exposure to moderately bright light (∼450 lux; ∼1.2 W/m^2^) for half of each of the scheduled wake episodes (second or first half, see Methods for details).

The period of the circadian timing system is genetically determined, involving transcription-translation feedback loops of clock genes [17], [18]. The period of the circadian pacemaker influences morning-eveningness preferences [19] and is important in the pathophysiology of certain circadian rhythm sleep disorders. Polymorphisms of clock genes such as Per2 and Per3 have been associated with advanced sleep phase disorder, delayed sleep phase disorder, and diurnal preference [20]–[23]. Furthermore, if a difference between the period of the circadian pacemaker and the period of the Earth's rotation cannot be corrected by daily time cues, this can lead to non-24-hour sleep-wake disorder, as often observed in completely blind individuals [24], [25]. Data in non-humans indicate that the near-24-h circadian period may be modified by prior environmental conditions, notably the period of the imposed light-dark cycle [26]. If the human circadian period would show such plasticity, this would have important implications for the treatment of circadian rhythm sleep disorders and for the development of countermeasures for human space exploration. We therefore tested the second hypothesis that the period of the circadian pacemaker in humans would be modified following entrainment to non-24-h day lengths, such that the intrinsic circadian period would be lengthened following entrainment to a longer-than 24-h day-night cycle (i.e., 24.65-h) as compared to the intrinsic circadian period following entrainment to a shorter-than 24-h day-night cycle (i.e., 23.5-h). We have measured the period of plasma melatonin, plasma cortisol, and core body temperature rhythms under controlled conditions of a forced desynchrony (FD) protocol to estimate intrinsic circadian period immediately following release from entrainment to each of these two day lengths [13].

## Results

**Figure 1:**
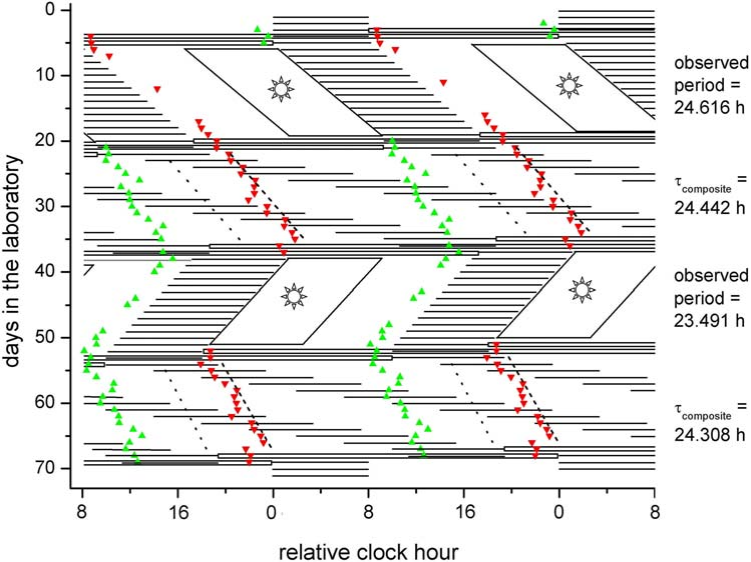
Raster plot of the study design for a representative individual (2478). The study protocol is double plotted such that consecutive days are next to and beneath the other. Scheduled sleep episodes in complete darkness (horizontal black lines), exposure to moderately bright light (parallelogram boxes with sun; part of wake episodes Days 6–19, 38–51), and CP procedures (open horizontal boxes; on Days 4–5, 20–21, 36–37, 52–53, 68–69) are indicated. Except for the scheduled wake episodes during the baseline (Day 1–3) and recovery days (Day 70–73; ∼90 lux [∼0.23 W/m^2^]) and exposure to moderately bright light (∼450 lux [∼1.18 W/m2]), lights were dim throughout the study (∼1.8 lux [∼0.0048 W/m^2^]). DLMOn_25% _(green upward-triangles) and DLMOff_25% _(red downward-triangles) are shown during dim light conditions. The intrinsic circadian period of the melatonin, cortisol, and temperature cycles during the FD protocols (Days 22–35, 54–67) were computed by a nonorthogonal spectral analysis technique. The fitted phase of the minimum of core body temperature (gray dotted line) and of the maximum of plasma cortisol (black dashed line) during the FD protocols are indicated. The observed circadian periods during the exposure to the 24.65-h day and to the 23.5-h day and the composite estimate of the intrinsic circadian period as computed by averaging the intrinsic period estimate from plasma melatonin, plasma cortisol, and core body temperature data (τ_composite_) [13] for both FD protocols are indicated to the right of the figure.

**Figure 2:**
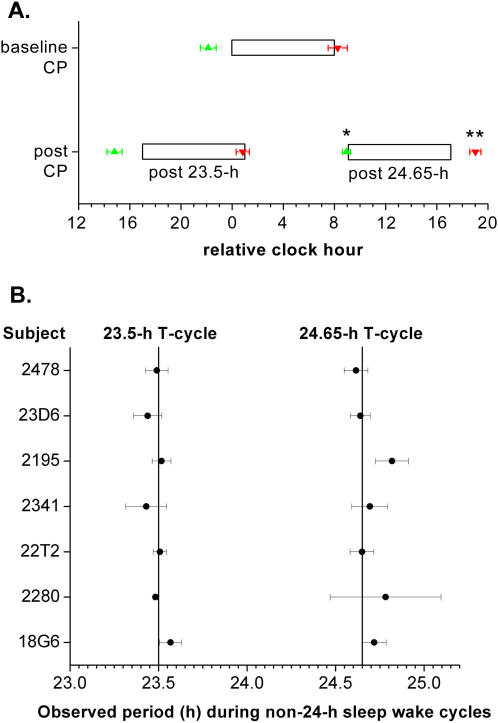
Lighting regime was effective for entraining circadian timing system to 24.65-h and 23.5-h rest-activity cycles. (A) The phase angles of entrainment were not different between the CP protocols at baseline and that following the 23.5-h days. The phase angles of entrainment were significantly different between the CP protocols at baseline and that following the 24.65-h days, such that the melatonin DLMOn_25%_ and DLMOff_25%_ during the CP protocol following the 24.65-h days relative to the scheduled sleep episode occurred even later than during the baseline CP protocol. Therefore, the lighting regimes were effective in maintaining entrainment to both non-24-h sleep-wake cycles at an appropriate phase angle. During the CP protocol following fourteen 23.5-h days (post 23.5-h), the timing of the scheduled sleep episode occurred 7 clock hours earlier than during the CP protocol preceding these shorter-than-24-h days. During the CP protocol following fourteen 24.65-h days (post 24.65-h), the timing of the scheduled sleep episode occurred 9.1 clock hours later than during the CP protocol preceding these longer-than-24-h days. Open bars, scheduled 8-h sleep episodes during CP protocols; green upward-triangle, DLMOn_25%_; red downward-triangle, DLMOff_25%_; error bars, SEM; *, P<0.01; **, P<0.005. (B) Light was effective for entraining the circadian timing system, such that the observed circadian period was similar to the period of the imposed non-24-hour sleep-wake cycles. Vertical lines, 23.5-h and 24.65-h day-night cycles (T-cycles); Error bars, 95% confidence interval.

Testing the first hypothesis, we found that the model-based lighting regimen were effective at entraining the melatonin rhythm to the 23.5-h and 24.65-h rest-activity cycles (Figure 1 and 2). The phase angles of entrainment between the 25% dim light melatonin onset (DLMOn_25%_) and bedtime and between the 25% dim light melatonin offset (DLMOff_25%_) and scheduled waketime during the constant posture (CP) protocol following the 23.5-h rest-activity cycles did not significantly change as compared to those phase angles during the baseline CP protocol (Figure 2A). This shows that the lighting regimen resulted in daily phase advances required to maintain entrainment to the 23.5-h rest-activity cycles. In fact, the DLMOn_25%_ relative to bedtime and the DLMOff_25%_ relative to scheduled waketime during the CP protocol following the 24.65-h light-dark cycles were significantly later by 1 h and 42 min (P<0.01) and 1 h and 40 min (P<0.05), respectively, as compared to their timing during the baseline CP protocol (Figure 2A). This shows that the lighting regimen resulted in even larger daily phase delays than required to maintain entrainment to the 24.65-h rest-activity cycle. Both lighting regimens furthermore maintained a phase angle of entrainment such that melatonin levels remained high during the scheduled sleep episodes and low during the scheduled wake episodes following the two-week 23.5-hour and two-week 24.65-hour sleep-wake cycle.

During the shorter-than-24-h rest-activity cycle, the observed period for the group was not different from the imposed 23.5-h cycle (23.49 h; 95% confidence interval [95% CI]: 23.46–23.53 h; Figure 2B). The 95% CI of the individual observed period overlapped 23.5 h for 5 subjects. For two subjects the estimate of observed period deviated by −1 or +4 min from 23.5 h (2280 and 18G6, respectively). During the longer-than 24-h rest-activity cycle, the observed period for the group was not different from the imposed 24.65-h cycle (24.70 h; 24.65–24.76 h). The 95% CI of the individual observed period overlapped 24.65 h for 5 subjects. For two subjects the estimate of observed period deviated by +4 or +10 min from 24.65 h (18G6 and 2195, respectively).

**Figure 3:**
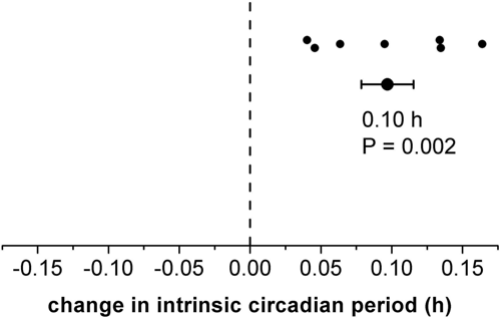
Plasticity of the human circadian period. Intrinsic circadian period was significantly longer following the 24.65-h day versus than following the 23.5-h day, as estimated during two-week FD protocols in dim light conditions. This was true when using plasma melatonin data (P = 0.002), plasma cortisol data (P = 0.004), core body temperature data (P = 0.02), and the composite [13] (P = 0.002) for period assessment, the later depicted here. There were no significant differences between melatonin, cortisol, and temperature period assessments. There was no significant order effect. Small filled circles; individual changes in intrinsic circadian period following exposure to both non-24-h day-night cycles (post 24.65–post 23.5); large filled circle; group difference; error bars, SEM.

Testing the second hypothesis, we found that the intrinsic circadian periods of the plasma melatonin (0.05±0.01 h; P = 0.002), plasma cortisol data (0.14±0.03 h; P = 0.004), core body temperature data (0.10±0.03 h; P = 0.02), and the composite [13] (0.10±0.02 h; P = 0.002), as assessed during the two-week FD protocol, were significantly longer following entrainment to the 24.65-h day than following entrainment to the 23.5-h day (Figures 1 and 3). There were no significant differences between melatonin, cortisol, and temperature period assessments when analyzed independently.

## Discussion

In this study, we developed a model-based lighting regimen that was effective for entraining individuals at a normal phase angle to the 24.65-h Martian sol and to a 23.5-h rest-activity cycle. Second, we showed for the first time plasticity of the human circadian timing system that regulates period, with prior entrainment to non-24-h rest-activity and light-dark cycles resulting in long-term changes in the intrinsic human circadian period (after-effects of entrainment).

We have developed a robust countermeasure composed of appropriately timed moderately bright light, dim light, and darkness, based on the human phase response curve, intensity response curve, and predictions from Kronauer's mathematical model of the effects of light on the human circadian pacemaker [5]–[7], [15], [16] to entrain at an appropriate phase angle the human circadian pacemaker to longer- and shorter-than 24-h day lengths. The findings of the current study validate the application of this mathematical model for the development of lighting countermeasures for entrainment to non-24-h sleep-wake schedules demanded by ground-based and flight-based space exploration missions [10], [14], [27]. Similarly, for circadian sleep rhythm disorders, where also differences between the intrinsic circadian period and the desired rest-activity cycle compromise restorative sleep and daytime cognitive functioning, the current findings may help design treatment of these disorders. Based on the spectral sensitivity of retinal photoreceptor systems involved in non-image-forming photic responses, additional research should be conducted to evaluate strategies to decrease further the light intensity or duration required by optimizing wavelength [8], [28]. Previous studies have reported lighting schemes capable of entraining some humans to non-24-h sleep-wake cycles [9], [29]–[31]. However, all but one of these studies did not show consistent circadian entrainment at a normal phase angle and/or used unreliable circadian phase markers (e.g., self-reported sleep-wake cycle or temperature phase estimates that were masked [32] under ambulatory conditions) [9], [29], [30]. A modulated light exposure composed of three different light levels during scheduled wakefulness, including two 45-min light pulses of 9,500 lux, was able to entrain subjects at a normal phase angle to a T-cycle 1h longer than each subject's intrinsic circadian period [31]. The current study extends these findings by showing that a simpler lighting regimen that includes 450 lux for half of the wake episode can also be effective for entraining subjects at a normal phase angle to a longer-than-24-h day and a shorter-than-24-h day.

Furthermore, here we show that entrainment to a non-24-h sleep-wake cycle can produce an “after-effect” on the intrinsic circadian period in humans and thus demonstrates plasticity of the human circadian timing system. Our finding of after-effects of entrainment in humans demonstrates a fundamental property of the circadian time keeping system in humans that is consistent with that observed in non-humans [26]. The observed after-effects of entrainment on the three different circadian phase markers, i.e., melatonin, cortisol, and core body temperature assessed during two-week FD protocols, support the hypothesis that the after-effects of entrainment measured in this study reflect plasticity of the master circadian pacemaker located in the SCN. Whether neural plasticity within the SCN plays a role in our current observations will require further animal experimental studies [33]. Another example of plasticity of the circadian timing system is the influence of prior light history on the sensitivity to a subsequent light exposure, which has been shown for melatonin suppression and phase shifting in rodents [34]. For humans, similar adaptation to preceding light intensity has been published for melatonin suppression [35]–[37]. Whether prior light history affects the phase shifting response in humans has yet to be shown. A potential underlying mechanism for adaptation of the circadian timing system to background light exposure is adaptation of the intrinsically photosensitive retinal ganglion cells, which drive circadian phase-shifting and melatonin suppression via the SCN [38]. It is unknown whether long-term depression of the electrical activity of SCN neurons following light exposure is involved in the observed adaptation of the circadian system to background light history [39].

The magnitude of the after-effects of entrainment in the current study (0.1 h), in which T-cycles that differed from 24 hours by 0.5 and 0.65 hours were imposed on the human subjects for 14 days, was smaller than that observed in studies performed in mice in which a gradually changing T-cycle that differed from 24 hours by ±4 hours was imposed for approximately 50 days [26]. Further research will be needed to determine whether the difference in the magnitude of the after-effects observed in these studies was due to species differences, the longer duration of exposure to the non-24-h T-cycle, or the larger differences in the preceding T-cycles in these animal experimental studies (20-h vs. 28-h T-cycles) as compared to the current human study (23.5-h vs. 24.65-h T-cycles). Moreover, entrainment to the more extreme T-cycles in the animal experimental studies was achieved by gradually lengthening or gradually shortening the T-cycle each day, which may have influenced the observed after-effects. Unfortunately, it is not known whether human subjects could be entrained to such extreme T-cycles. Furthermore, it would have required 145 days for each subject studied to duplicate the earlier mouse study,, which would make such studies difficult to conduct in humans. After-effects typically show a slow decay back to the intrinsic circadian period over the course of many cycles; for example, in mice and sparrow, after-effects on circadian period have been reported to persist for ∼100 cycles [26], [40]. The two-week FD protocols on which the period estimates were based in the current study were too short to assess the time course of this decay in humans.

Our finding of an after-effect of entrainment on circadian period may help to explain, as least in part, the observed difference in average circadian period between sighted individuals (∼24.2 h) and blind individuals not entrained to the 24-h day while living in society (∼24.5 h), although selection bias and other factors may play an additional role in the longer average period reported in non-entrained blind individuals [13], [41]. This explanation is corroborated in non-human mammals, in which the observed circadian period upon release from entrainment to the 24-h day has been reported to be closer to 24.0 h than following exposure to constant darkness for an extended time or after blinding [42], [43].

Also photoperiod, the fraction of the light episode relative to the dark episode within a light-dark cycle, has been reported to affect circadian period in mice and birds, with longer light episodes shortening circadian period. Such differences could account for seasonal variations in circadian timekeeping [26], [40], [44]. In animal studies, it has been reported that even a single light exposure can result in changes in the intrinsic circadian period that are comparable in magnitude to after-effects of entrainment [26], [40], [42]. If this were also the case in humans, then even a single transmeridian flight (rapid travel across multiple time zones), with a sudden shift in the timing of the light-dark cycle, could induce changes in intrinsic circadian period. Such post-travel after-effects in circadian period may affect the 50–100 million people who experience jet travel each year. Similarly, these results suggest that repeated shifts in the sleep-wake cycle and the circadian timing system in shift workers may result in recurrent and longer-term changes in circadian period. Additional research is needed to investigate long-term after-effects of travel across time zones and shift work on human circadian period.

The findings of circadian entrainment to non-24-h sleep-wake rhythms and after-effects on intrinsic circadian period following entrainment have important implications for the development of treatments for circadian rhythm sleep disorders, such as delayed sleep phase disorder and advanced sleep phase disorder, in which circadian sleep drive occurs much later or earlier than desired, respectively. These findings may also influence development of countermeasures for human adaptation to non-24-h-sleep-wake schedules, as encountered by shift-workers, in jet lag, in submarine naval operations, and during short-term and long-term human space exploration [10], [20], [21], [45]. The relative contribution of SCN and extra-SCN tissue in after-effects of entrainment will require further study [46]. Current genetic or neural plasticity models do not include a mechanism that explains after-effects of entrainment. Understanding the molecular and neural basis of after-effects of entrainment could allow for a targeted strategy to optimize further treatment of circadian rhythm sleep disorders and human space exploration.

## Methods

### Subjects

Seven healthy young men (age 22–40 yrs) completed the 73-day in-laboratory studies. Subjects were free from medications and drugs for the week prior and during the study. Subjects reported no shift work for three years nor crossing more than one time zone for three months prior to study. Subjects maintained a regular 8-h sleep and 16-h wake schedule while living at home for three weeks prior to study, as verified by sleep log, call-in times, and wrist actigraphy (Actiwatch, Minimitter, Bend, OR). Subjects gave written informed consent and the protocol was approved by the Partners Human Research Committee.

### In laboratory condition

Entrainment to non-24-h day lengths and after-effects of entrainment on period were tested in a randomized, within-subject, crossover design. Subjects lived in a personal laboratory room free of time cues for 73 days. Ambient light and room temperature were controlled and sleep opportunities and meals were scheduled. Ceiling-mounted fluorescent lamps (Philips, Eindhoven, The Netherlands) with a 4100-K color temperature produced a spectrum of white light. Napping and exercise were proscribed. After admission to the laboratory, subjects were maintained on a 24-h sleep-wake cycle for three days (Figure 1). This was followed by a 40-h CP protocol during which the subjects were scheduled to sleep at their habitual times but maintained a semi-recumbent posture in dim light conditions (0 lux during scheduled sleep and ∼1.8 lux [∼0.0048 W/m^2^] during wakefulness) thereby allowing accurate assessment of intrinsic melatonin phase. After the CP protocol, subjects were scheduled to a 24.65-h or 23.5-h day for 14 cycles, maintaining a 2∶1 wakefulness:bedrest ratio. Given that the resetting effects of light are both phase-dependent [7], [15] and intensity-dependent [5], [6], we used Kronauer's mathematical model [16] to design a temporal pattern of environmental light exposure that would facilitate entrainment to the Martian sol and to a 23.5-h day (Figure 1) with light exposure following the circadian core body temperature minimum (i.e., morning) causing phase advances and with light exposure preceding the circadian core body temperature minimum (i.e., evening) causing phase delays. During each of the 23.5-h and 24.65-h days, lights were moderately bright (∼450 lux [∼1.18 W/m^2^]) for approximately half of scheduled wakefulness (the first and the second half, respectively), and dim (∼1.8 lux) for the remainder of scheduled wakefulness. The entrainment schedule was followed by a second 40-h CP protocol and a two-week FD protocol, composed of 28.0-h dim light–dark (∼1.8 lux-0 lux) activity–rest cycles, that was used to estimate the intrinsic circadian period by assessing the subjects' plasma melatonin, plasma cortisol, and core body temperature rhythms [13]. The duration of the wake episode of the third CP protocol was adjusted for each subject to realign the sleep-wake cycle for the second half of the study such that the phase angle between the core body temperature minimum and scheduled waketime at the end of the third CP protocol would be similar to that during the first (baseline) CP protocol. Following the third CP protocol, subjects were entrained to the other non-24-h cycle (23.5-h or 24.65-h, respectively), again followed by a fourth CP protocol, circadian period assessment during a two-week 28-h FD protocol, and a fifth CP protocol. Following the fifth (final) CP protocol, subjects were scheduled according to their habitual sleep-wake schedule for three days to facilitate reentrainment to their habitual sleep-wake cycle (Figure 1).

### Analysis

Blood was sampled by an intravenous catheter at hourly intervals throughout the FD protocols and CP protocols, and for three 2–3-day sampling windows during the 23.5-h and 24.65-h entrainment cycles. Plasma melatonin and cortisol levels were assayed via radioimmunoassay I^125^ (Pharmasan Laboratories, Osceola, WI, USA). The sensitivity of the melatonin assay was 0.7 pg/mL and the interassay coefficient of variation was 13.2% and 8.4% at a mean concentration of 17.3 and 69 pg/mL, respectively. The sensitivity of the cortisol assay was 0.11 µg/dL and the interassay coefficient of variation was 7.9% and 6.8% at a mean concentration of 5.1 and 18.5 µg/dL, respectively. Core body temperature was recorded continuously during the two FD protocols and the five CP protocols every minute via a rectal thermister (Yellow Springs Instrument Company, Yellow Springs, OH USA).

To distinguish definitively between entrainment and relative coordination, a much longer inpatient study (>6–12 months) on each non-24-h rest-activity cycle would have been required. We have therefore used a classification method of entrainment that is consistent with that used in our prior research [9], [14] and with that from studies of blind individuals [47], [48]. Using this classification, entrainment to the non-24-h sleep-wake cycles was defined as the 95% confidence interval (95% CI) of the observed circadian period overlapping with the period of the imposed rest-activity cycle. In addition, we tested whether subjects were entrained at a normal phase angle, which was defined as a similar phase angle of entrainment (i.e., timing of circadian timing system relative to sleep-wake cycle) observed following the non-24-h-sleep wake cycle as that observed at baseline (T = 24 h) and tested with a 2-tailed t test for dependent samples [9], [14]. The observed circadian period was estimated by linear regression of the DLMOn_25%_ and DLMOff_25%_ times during the 23.5-h day-night cycle and the 24.65-h day-night cycle, respectively, after exclusion of the first 5 cycles, to minimize transient adjustments to imposed T cycles. The phase angle of entrainment was assessed by comparing the timing of the DLMOn_25%_ and DLMOff_25%_ relative to the scheduled bedtime and scheduled waketime, respectively.

For assessment of the after-effects of entrainment, the intrinsic circadian period following the 24.65-h days and following the 23.5-h days were compared and tested with a 2-tailed t test for dependent samples. Circadian period estimates were based on plasma melatonin, plasma cortisol, and core body temperature data collected during FD and were computed by a nonorthogonal spectral analysis technique with an exact maximum likelihood fitting procedure [13]. The composite estimate of the intrinsic period for each subject was computed by averaging the intrinsic circadian period estimates for plasma melatonin, plasma cortisol, and core body temperature data [13]. Please refer to Text S1 for supplementary materials and methods.

## Supporting Information

Text S1 Supplementary materials and methods
(0.04 MB DOC)

Alternative Language Abstract S1 Translation of Abstract into Dutch
(0.03 MB DOC)
