# Supplementary material for: Plasticity of the Intrinsic Period of the Human Circadian Timing System
Source: PLoS One. 2007 Aug 8;2(8):e721. doi: 10.1371/journal.pone.0000721 (PMC1934931; doi:10.1371/journal.pone.0000721)
Supplement: Text S1 — Supplementary materials and methods (0.04 MB DOC) [file pone.0000721.s001.doc]

Plasticity of the Intrinsic Period of Human Circadian Timing System

**Frank A.J.L. Scheer,^*^ Kenneth P. Wright Jr.,^†^ Richard E. Kronauer, Charles A. Czeisler**

*Division of Sleep Medicine, Department of Medicine, Brigham & Women’s Hospital and Division of Sleep Medicine, Harvard Medical School, Boston*

*to whom correspondence should be addressed.

†Present address: Sleep and Chronobiology Laboratory, Department of Integrative Physiology, Center for Neuroscience, University of Colorado, Boulder, CO

This document provides supplementary information for the above article. We provide more details for some of the methods applied in the presented studies.

**Subjects**

The initial aim was to study an equal number of males and females. Since only one female was studied and we therefore did not have the opportunity to evaluate the interaction of the menstrual cycle with the 73-day protocol, we present only the data from the males here. The data of seven young men (age 22-40 yrs) were included in the analysis. Subjects were healthy, as established by medical history and physical and psychological examination. All subjects were free of medication, drugs, and of caffeine, nicotine, and alcohol for the week prior and during the study.

**In laboratory condition**

The lighting regimen during the first three days after admission to the laboratory was similar as published before [1]. During all scheduled sleep episodes, all lights were off (0 lux). During the Constant Posture (CP) protocols and the Forced Desynchrony (FD) protocols, lights were dim during scheduled wake episodes (~1.8 lux [~0.0048 W/m^2^] at 137 cm in the horizontal angle of gaze and <8 lux [0.025 W/m^2^] at 187 cm in the vertical angle). During each of the 23.5-h and 24.65-h days, lights were moderately bright (~450 lux [~1.18 W/m^2^] at 137 cm in the horizontal angle of gaze) for approximately half of scheduled wakefulness (the first and the second half, respectively), and dim (~1.8 lux) for the remainder of scheduled wakefulness.

During the 24.65-h days, subjects were scheduled to 16 h and 26 min of wakefulness and 8 h and 13 min of sleep. During the 23.5-h days, subjects were scheduled to 15 h and 40 min of wakefulness and 7 h and 50 min of sleep. During the 28-h FD days, subjects were scheduled to 18 h and 40 min of wakefulness and 9 h and 20 min of sleep. Two 2-week Forced Desynchrony protocols (FD) were used to estimate the intrinsic circadian period of individuals by assessing their plasma melatonin, plasma cortisol, and core body temperature rhythms [2]. The FD protocol is based on two principles: 1) to minimize any masking effects of circadian time cues; and 2) to uniformly distribute any remaining time cues across the circadian cycle. Applying principle 1, lights were maintained at a low intensity to minimize the phase-shifting influence of light on the circadian timing system during the FD protocols. Applying principle 2, the sleep-wake cycle (including changes in behavioural activity, body posture, showers, and meals) was constrained to a period outside of the range of entrainment for the human circadian pacemaker (28-hours) under such dim light conditions during the FD protocols such that any non-photic time cues were uniformly distributed across the circadian cycle.

**Analysis**

Melatonin onset and offset were defined by the linearly interpolated point in time at which the melatonin concentrations increased and decreased, respectively, to reach the 25% of the fitted peak-to-trough amplitude of the 3-harmonic of each individual’s data from the first CP protocol. This 25% dim light melatonin onset (DLMO_25%_) and offset (DLMOff_25%_), as determined during the first CP protocol was applied to data at all other portions of the protocol.

**References**

1. Wright Jr. KP, Hughes RJ, Kronauer RE, Dijk DJ, Czeisler CA (2001) Intrinsic near-24-hour pacemaker period determines limits of circadian entrainment to a weak synchronizer in humans. Proc Natl Acad Sci USA 98: 14027-14032.

2. Czeisler CA, Duffy JF, Shanahan TL, Brown EN, Mitchell JF, Rimmer DW, Ronda JM, Silva EJ, Allan JS, Emens JS, Dijk DJ, Kronauer RE (1999) Stability, precision, and near-24-hour period of the human circadian pacemaker. Science 284: 2177-2181.
