# Supplementary material for: Plasticity of the Intrinsic Period of the Human Circadian Timing System
Source: PLoS One. 2007 Aug 8;2(8):e721. doi: 10.1371/journal.pone.0000721 (PMC1934931; doi:10.1371/journal.pone.0000721)
Supplement: Alternative Language Abstract S1 — Translation of Abstract into Dutch (0.03 MB DOC) [file pone.0000721.s002.doc]

**Plasticiteit van de Intrinsieke Periode van de Menselijke Biologische Klok**

Bemande Mars expidities zullen aanpassing vereisen aan de 24.65-urige Mars dag-nacht cyclus (sol), waaraan de menselijke circadiane pacemaker zich niet kan synchroniseren onder licht intensiteiten waaraan astronauten typisch worden blootgesteld. Onvermogen van het circadiane systeem om te synchroniseren aan de gewenste rust-activiteits cyclus leidt tot verstoorde slaap en verminderd cognitief functioneren. Bovendien vormen verschillen tussen de intrinsieke circadian periode (cyclus lengte) en de 24-uurs light-donker cyclus op aarde een onderliggende oorzaak van slaap-waak ritme stoornissen bij de mens, zoals een vervroegde of verlate slaapfase of een onregelmatig slaappatroon zonder duidelijk 24-uurs ritme. Ten eerste hebben we daarom getest of blootstelling aan een licht-schema gebaseerd op model berekeningen de menselijke circadiane pacemaker met een normale fase-relatie kan synchroniseren aan de 24.64-urige Mars sol en aan de 23.5-urige dag lengte vaak vereist van astronauten gedurende kort-durende ruimte vluchten. Ten tweede hebben we getest of synchronisatie aan niet-24-urige light-donker cycli vervolgens leidt tot modificatie van de intrinsieke periode van het circadiane systeem in de mens.

We laten hier zien dat blootstelling aan gematigd helder licht (~450 lux; ~1.2 W/m2) gedurende de tweede of eerste helft van de ingeplande waak periode effectief is om individuen te synchroniseren aan de 24.65-urige Mars sol en aan een 23.5-urige dag lengte, respectievelijk. Schattingen van de circadiane periode op basis van plasma melatonine, plasma cortisol, en kern lichaams temperatuur gemeten tijdens geforceerde desynchronisatie-protocollen openbaarde dat de intrinsieke circadiane periode van de menselijke circadiane pacemaker significant langer was na synchronisatie aan de Mars sol in vergelijking met na synchronisatie aan de 23.5-urige dag.

Deze laatste bevinding van een na-effect van synchronisatie onthuld voor het eerst plasticiteit van de periode van het menselijke circadian systeem. Beide bevindingen hebben belangrijke implicaties voor de behandeling van slaap-waak ritme stoornissen en bemande ruimtevaart.
